# Silver-dotted titanium dioxide nanocomposites for highly efficient photo-induced enhanced Raman spectroscopy in trace detection of weak-Raman-response molecules

**DOI:** 10.1039/d5ra05258k

**Published:** 2025-10-29

**Authors:** Quan-Doan Mai, Dang Thi Hanh Trang, Vo Thi Le Na, Ta Ngoc Bach, Ong Van Hoang, Nguyen Thi Thanh Tuyen, Nguyen Duy Hung, Anh-Tuan Pham, Anh-Tuan Le

**Affiliations:** a Phenikaa University Nano Institute (PHENA), Phenikaa School of Engineering (PSE), Phenikaa University Hanoi 12116 Vietnam doan.maiquan@phenikaa-uni.edu.vn fattuan@phenikaa-uni.edu.vn tuan.leanh@phenikaa-uni.edu.vn; b Faculty of Materials Science and Engineering, Phenikaa School of Engineering (PSE), Phenikaa University Hanoi 12116 Vietnam; c Institute of Materials Science (IMS), Vietnam Academy of Science and Technology 18 Hoang Quoc Viet Hanoi 10000 Vietnam; d University of Transport Technology Trieu Khuc, Thanh Xuan District Hanoi Vietnam; e Faculty of Electronic Materials and Devices, Ha Noi University of Science and Technology (HUST) 01 Dai Co Viet HaNoi 10000 Vietnam

## Abstract

Surface-enhanced Raman spectroscopy (SERS) enables highly sensitive molecular detection, including single-molecule analysis, for species with strong Raman scattering. However, conventional SERS platforms often fail to detect analytes with inherently weak Raman responses, limiting their applicability in trace sensing of these molecules. Here, we present photo-induced enhanced Raman spectroscopy (PIERS) as an advanced SERS technique based on silver-dotted titanium dioxide (Ag-dot TiO_2_) nanocomposite (NC) substrates, which synergistically integrate plasmonic Ag nanoparticles with photo-activated TiO_2_ to significantly amplify Raman signals beyond the capability of conventional SERS. Congo red (CR), a bulky dye molecule with low Raman activity, was selected as the model analyte. The PIERS signal exhibited an 8.9-fold enhancement over standard SERS with high reliability. The enhancement was strongly dependent on both the pre-irradiation duration and recovery time of the Ag-dot TiO_2_ substrates, highlighting the tunable nature of the PIERS effect. To further assess its versatility, the PIERS platform was tested on thiram (a pesticide) and urea (a small biomolecule) and demonstrated reliable trace-level detection in both cases. These obtained results establish Ag-dot TiO_2_-based PIERS platform as a promising approach for ultrasensitive detection of weak-Raman-response molecules, with broad implications for environmental monitoring, food safety, and biomedical diagnostics.

## Abstract 2

In this work, a photo-induced enhanced Raman spectroscopy (PIERS) platform based on Ag-dotted TiO_2_ nanocomposites was developed to overcome the limitations of conventional SERS in detecting weak-Raman-response molecules.

## Graphical abstract

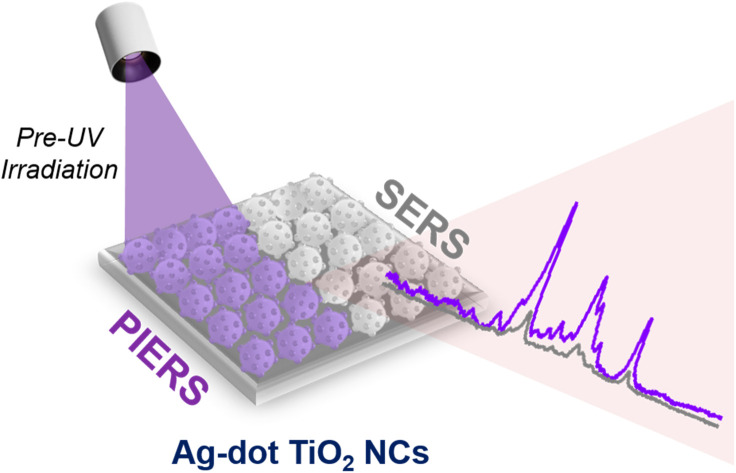

## 1 Introduction

Surface-enhanced Raman spectroscopy (SERS) has emerged as one of the most powerful and widely applied analytical techniques, offering distinct advantages over other sensing methods. These include the ability to provide detailed molecular fingerprint information through vibrational signatures and the capacity for single-molecule detection.^1–3^ The enhancement in SERS originates from the three-body interaction among light, analyte molecules, and noble-metal nanostructures (or substrates), where molecules adsorbed on a roughened metal surface experience a dramatic amplification of their Raman signals.^4,5^ Two primary mechanisms – electromagnetic (EM) enhancement and chemical (CM) enhancement – are widely accepted as the dominant contributors to this signal amplification.^2,6^ When noble-metal structures are scaled down to the nanometer level, not only does the adsorption efficiency increase due to the larger surface area and more active sites, but the localized surface plasmon resonance (LSPR) generated by metal nanoparticles also creates extremely intense electromagnetic fields, leading to substantial SERS signal enhancement.^7^ Driven by advancements in nanoscience, numerous nanostructure-based SERS substrates have been developed over the past decades, enabling ultra-sensitive detection of important targets such as dyes, pesticides, explosives, and biomarkers – molecules that are critical in areas ranging from environmental safety to biomedical diagnostics.^8–10^ However, despite these advances, a significant limitation remains: conventional SERS platforms often fail to effectively detect molecules with weak Raman responses, even at high concentrations. Small molecules with low Raman cross-sections or those with bulky, sterically hindered structures frequently yield insufficient Raman signals, posing a major challenge for trace-level detection.^11–13^ Overcoming this limitation is essential for expanding the practical applicability of SERS in real-world sensing scenarios.

Recently, several advanced SERS strategies have been developed to further improve sensitivity compared with conventional SERS. For example, oxygen incorporation or extraction during substrate fabrication can significantly enhance SERS signals compared with oxygen-untreated substrates;^14^ surface hydrogenation engineering can induce amorphous surface layers that improve SERS activity;^15^ and photo-induced enhanced Raman spectroscopy (PIERS) based on pre-UV-irradiated substrates can create temporary oxygen vacancies that facilitate charge transfer and amplify Raman signals.^16^ Among these approaches, PIERS has attracted increasing attention because it demonstrates effective enhancement for a wide variety of analytes, including those with intrinsically weak Raman responses.^16^ PIERS enhances Raman signal intensity by employing photo-activated metal/semiconductor hybrid substrates that undergo ultraviolet (UV) pre-irradiation. This process generates oxygen vacancies in the semiconductor component (*e.g.*, TiO_2_, ZnO, WO_3_), introducing mid-gap states that facilitate more efficient charge transfer at the metal–semiconductor-analyte interfaces.^16,17^ First introduced by Ben-Jaber *et al.* in 2016, the PIERS phenomenon was demonstrated on Au/TiO_2_ substrates pre-irradiated with 254 nm UV light for 4 hours. The technique successfully improved Raman signals across various molecular systems, including small and low Raman cross-section species such as dinitrotoluene, cyclotrimethylenetrinitramine, and glucose.^18^ Subsequently, PIERS has been further explored with a variety of substrate structures and analytes.^16^ Man *et al.* reported a 3-fold enhancement over conventional SERS using Ag nanoparticles on TiO_2_ (anatase phase, thin-film) substrates pre-irradiated with 365 nm UV light for 30 minutes when detecting the small biomolecule adenosine triphosphate.^19^ Similarly, Barbillon *et al.* observed a 7.52-fold PIERS enhancement using Au nanoparticles on ZnO films pre-treated with 254 nm UV light for 30 minutes in the detection of thiophenol (a small molecule).^20^ Ren *et al.* reported an 8.1-fold PIERS enhancement for rhodamine 6G on substrates composed of Ag nanoparticles deposited on lithium niobate on insulator (LNOI).^21^ These studies collectively demonstrate that the PIERS effect offers substantial sensing enhancement, and its performance is highly dependent on both the design of the metal/semiconductor substrate and the nature of the target analyte. This makes PIERS a promising strategy for overcoming the intrinsic limitations of conventional SERS, especially for molecules with poor Raman activity.

Weak-Raman-response molecules encompass not only small species but also bulky, structurally complex compounds that pose significant challenges for conventional SERS platforms. To broaden the applicability of promising techniques like PIERS, it is therefore essential to explore their performance in detecting this wider class of analytes. In this study, we investigate the PIERS activity of a nanostructured substrate composed of silver-dotted rutile-phase titanium dioxide nanoparticles (Ag-dotted TiO_2_ nanocomposites, or Ag-dot TiO_2_ NCs), using bulky Congo red (CR) with a weak Raman response as a representative analyte. The presence of photo-activatable TiO_2_ nanoparticles enables the application of the PIERS technique on the Ag-dot TiO_2_ NCs. PIERS experiments were conducted on Ag-dot TiO_2_ NCs pre-treated with UV irradiation at 365 nm for various durations prior to SERS measurement, and signal intensity was evaluated at different recovery times after the pre-irradiation process. The results reveal a strong dependence of signal enhancement on both UV pre-treatment and recovery time. The enhancement mechanism of PIERS over conventional SERS on Ag-dot TiO_2_ NCs, primarily attributed to the charge-transfer-based CM mechanism, is also discussed. Under optimal conditions, the Ag-dot TiO_2_ NCs deliver up to an 8.9-fold enhancement compared to standard SERS, achieving a detection limit as low as 5.32 × 10^−11^ M for CR – far surpassing previously reported SERS performances. To validate the broader applicability of this platform, we extended the PIERS evaluation to thiram (a pesticide) and urea (a small biomolecule), achieving trace detection limits of 1.89 × 10^−10^ M and 2.32 × 10^−7^ M, respectively. These results highlight the strong sensing capabilities of PIERS based on Ag-dot TiO_2_ NCs, offering great potential for trace detection of weak-Raman-response molecules – both bulky and small – in fields such as environmental monitoring, food safety, and biomedical diagnostics.

## 2 Materials and methods

### 2.1 Materials

The precursors, including tetrapropylammonium bromide (C_12_H_28_NBr, 99.9%), silver nitrate (AgNO_3_, ≥99.0%), sodium borohydride (NaBH_4_, 99%), and ethanol (C_2_H_5_OH, 98%), as well as the analytes including Congo red (C_32_H_22_N_6_Na_2_O_6_S_2_, >98.0%), thiram (C_6_H_12_N_2_S_4_, >95.0%), and urea (CH_4_N_2_O, 99%), were purchased from Shanghai Chemical Reagent Co. All chemicals were used as received. Titanium foils (purity: 99.9%) were purchased from Xi'an HST Metal Material Co., Ltd. and cut into dimensions of 50 mm × 10 mm × 1 mm. Double-distilled water was used throughout the experiments.

### 2.2 Synthesis of Ag-dotted TiO_2_ nanocomposite materials and their characterizations

The Ag-dotted TiO_2_ nanocomposites (Ag-dot TiO_2_ NCs) were synthesized through a two-step process. Initially, rutile-phase TiO_2_ nanoparticles were fabricated *via* an electrochemical approach, followed by the *in situ* formation of silver nanoparticles on their surfaces through a chemical reduction method. The electrochemical synthesis of TiO_2_ nanoparticles was conducted according to our previously reported method.^22^ Briefly, the setup consisted of a DC power source, two parallel titanium electrodes, and an aqueous electrolyte solution containing 1% tetrapropylammonium bromide (TPAB). A reaction beaker was prepared with 200 mL of distilled water and TPAB. The titanium electrodes were placed 3 cm apart and immersed in the electrolyte solution. A constant voltage of 12 V was applied for 5 hours at room temperature to initiate the electrochemical formation of colloidal TiO_2_, which appeared as a bright white suspension. The resulting colloidal TiO_2_ solution was then dried at 60 °C for 8 hours in an oven to evaporate the water, yielding an amorphous TiO_2_ powder. This powder was subsequently calcined at 800 °C to convert it into crystalline rutile-phase TiO_2_. To synthesize the Ag-dot TiO_2_ NCs, the obtained TiO_2_ powder was dispersed in 50 mL of distilled water using ultrasonic treatment and continuous magnetic stirring. A small amount of cetyltrimethylammonium bromide (CTAB) was added as a surfactant to improve the dispersion stability of TiO_2_ nanoparticles and to assist in controlling the spatial distribution of Ag^+^ ions near the TiO_2_ surface. Since CTAB plays an important role in promoting the nucleation and anchoring of Ag nanoparticles on TiO_2_ surfaces, three synthesis conditions were investigated: (i) without adding CTAB, (ii) with 1 mL of 0.01 M CTAB solution, and (iii) with 1 mL of 0.1 M CTAB solution. A predetermined amount of AgNO_3_ dissolved in 10 mL of distilled water was slowly added to the dispersion and stirred for 1 hour to ensure sufficient interaction between Ag^+^ ions and the TiO_2_ surface. Subsequently, an aqueous solution of NaBH_4_ was gradually introduced to chemically reduce Ag^+^ to metallic Ag^0^, leading to the formation of Ag nanoparticles directly on the TiO_2_ surfaces. The reaction was maintained under continuous stirring for 3 hours. The final product was collected by centrifugation and washed three times with ethanol to remove impurities. The cleaned material was then dried at 60 °C to obtain the Ag/TiO_2_ nanocomposite powders. As a result, three distinct Ag/TiO_2_ nanocomposites were obtained, differing in their structural characteristics depending on the presence and concentration of CTAB during synthesis. Their morphologies and SERS performances were subsequently examined to evaluate the effect of CTAB concentration on the structural formation and enhancement behavior of the Ag/TiO_2_ nanocomposites. The morphology of the TiO_2_ and Ag-dot TiO_2_ NCs was examined using field emission scanning electron microscopy (FE-SEM, Hitachi S-4800) operated at an accelerating voltage of 5 kV. Raman spectroscopy (Horiba Macro-RAM™) with a 785 nm laser was employed to analyze molecular vibrations and structural characteristics. Additionally, photoluminescence (PL) spectroscopy with an excitation wavelength of 380 nm was used to assess the optical properties of the materials. The crystalline characteristics of the Ag-dot TiO2 NCs was examined by X-ray diffraction (XRD) using a Bruker D5005 diffractometer equipped with Cu Kα radiation (*λ* = 1.5406 Å), operated at 40 kV and 30 mA.

### 2.3 Substrate preparation, SERS measurements

**Scheme 1:**
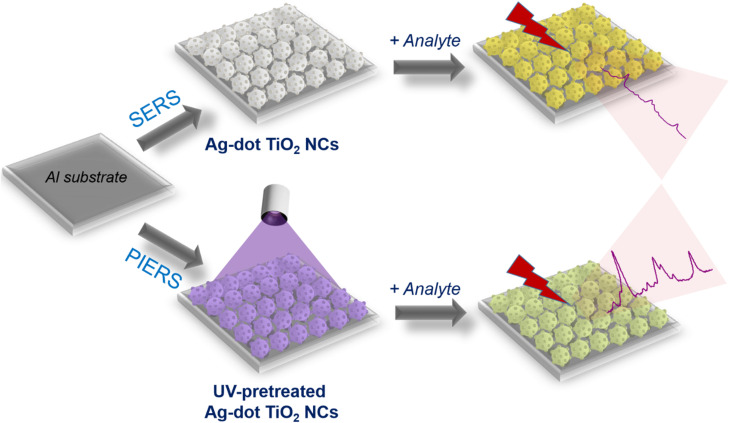
Experimental procedures for SERS and PIERS signal acquisition on Ag-dot TiO_2_ NC substrates.

Aluminum (Al) substrates with dimensions of 1 × 1 × 0.1 cm were prepared for the deposition of Ag-dot TiO_2_ nanocomposites (NCs) to serve as sensing platforms. A circular active area with a diameter of 0.2 cm was defined on the Al surface to receive both the materials and the analytes. The substrates were cleaned with ethanol and air-dried at room temperature (RT). The Ag-dot TiO_2_ NCs were dispersed in distilled water and drop-cast onto the designated active area of the Al substrates, followed by natural evaporation of the solvent. Analyte solutions were prepared at various concentrations: CR (10^−3^–10^−11^ M), thiram (10^−3^–10^−10^ M), and urea (10^−3^–10^−7^ M), using a serial dilution method with tenfold decrements. Due to its poor solubility in water, thiram was first dissolved in 1 mL of ethanol as a co-solvent before adding 9 mL of double-distilled water to reach the desired concentration of 10^−4^ M. A fixed volume of 5 μL of each analyte solution was drop-cast onto the Ag-dot TiO_2_ NC-coated area and allowed to dry naturally at RT. SERS measurements were performed using a MacroRamam™ spectrometer (Horiba) with a 785 nm laser as the excitation source (Scheme 1). The measurements employed a 100× objective lens with a numerical aperture of 0.90. The laser power was set at 45 mW and directed at a 30^°^ incident angle, resulting in a diffraction-limited laser spot diameter of 1.1 μm (calculated by 1.22*λ*/NA) and a focal depth of 115 nm. Each spectrum was recorded with a single 30-second exposure, and baseline correction was applied to the final data.

### 2.4. PIERS preparation and measurements

The Ag-dot TiO_2_ NC substrates were prepared using the same protocol for both SERS and PIERS experiments. For PIERS, however, the substrates underwent a UV pre-irradiation treatment at 365 nm before Raman signal acquisition (Scheme 1). The selection of 365 nm (∼3.40 eV) was based on its proximity to the band-gap excitation energy of rutile TiO_2_ (∼3.0 eV), which effectively induces surface oxygen vacancies at an appropriate rate while avoiding the potential structural degradation that may occur under higher-energy UV irradiation. The UV source was positioned 10 cm above the substrate surface, providing a suitable balance between avoiding thermal effects from excessive UV intensity and ensuring sufficient photon flux for stable photoactivation. In addition, the UV pre-irradiation process for PIERS activation was performed under standard laboratory atmospheric conditions to ensure the practical applicability of PIERS sensing as a facile and straightforward analytical protocol. The PIERS enhancement effect was evaluated by varying the pre-irradiation durations (5, 10, 20, 30, 40, 50, 60, and 80 minutes), followed by SERS measurements. In addition, the recovery time – *i.e.*, the time over which the PIERS effect persisted after stopping the UV exposure – was investigated by collecting Raman signals at 5, 10, 20, 30, 40, and 50 minutes post-irradiation. The optimal pre-irradiation time and recovery time were then applied to subsequent sensing procedures to assess the sensitivity and reliability of detecting CR, thiram, and urea molecules.

### 2.5. Calculation of limit of detection (LOD)

The limit of detection (LOD) was estimated from the calibration curve obtained through SERS measurements, using the Raman intensity of CR, thiram, and urea powders as reference signals. The LOD was determined using the following equation:^23^

1 LOD = 10^[(*Y*_average_+3SD)/*Y*_average_−*A*]/*B*^

In this context, *Y*_average_ refers to the mean Raman intensity calculated from ten successive measurements of CR, thiram, or urea in its solid state. The standard deviation (SD) was determined using these ten data points, following the equation shown below. The coefficients *A* and *B* represent the intercept and slope, respectively, of the linear regression obtained by plotting the logarithm of the SERS intensity (*y*) against the logarithm of the analyte concentration (*x*), as described by the equation: *y* = *A* + *B* × *x*.

SD was calculated according to the standard statistical method provided below.2
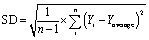
Here, *n* = 10 indicates the number of repeated measurements. *Y*_*i*_ corresponds to the Raman intensity obtained in the ith measurement, while *Y*_average_ represents the mean Raman signal derived from ten replicate measurements of solid-state CR, thiram, or urea.

To assess measurement repeatability and reproducibility, the relative standard deviation (RSD) was calculated using the conventional statistical formula shown below:3
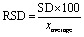
where SD refers to the standard deviation determined from eqn (2), and *x*_average_ indicates the average SERS intensity derived from multiple individual measurements.

## 3 Results and discussion

### 3.1. Characterizations of Ag-dotted TiO_2_ nanocomposite materials

**Fig. 1:**
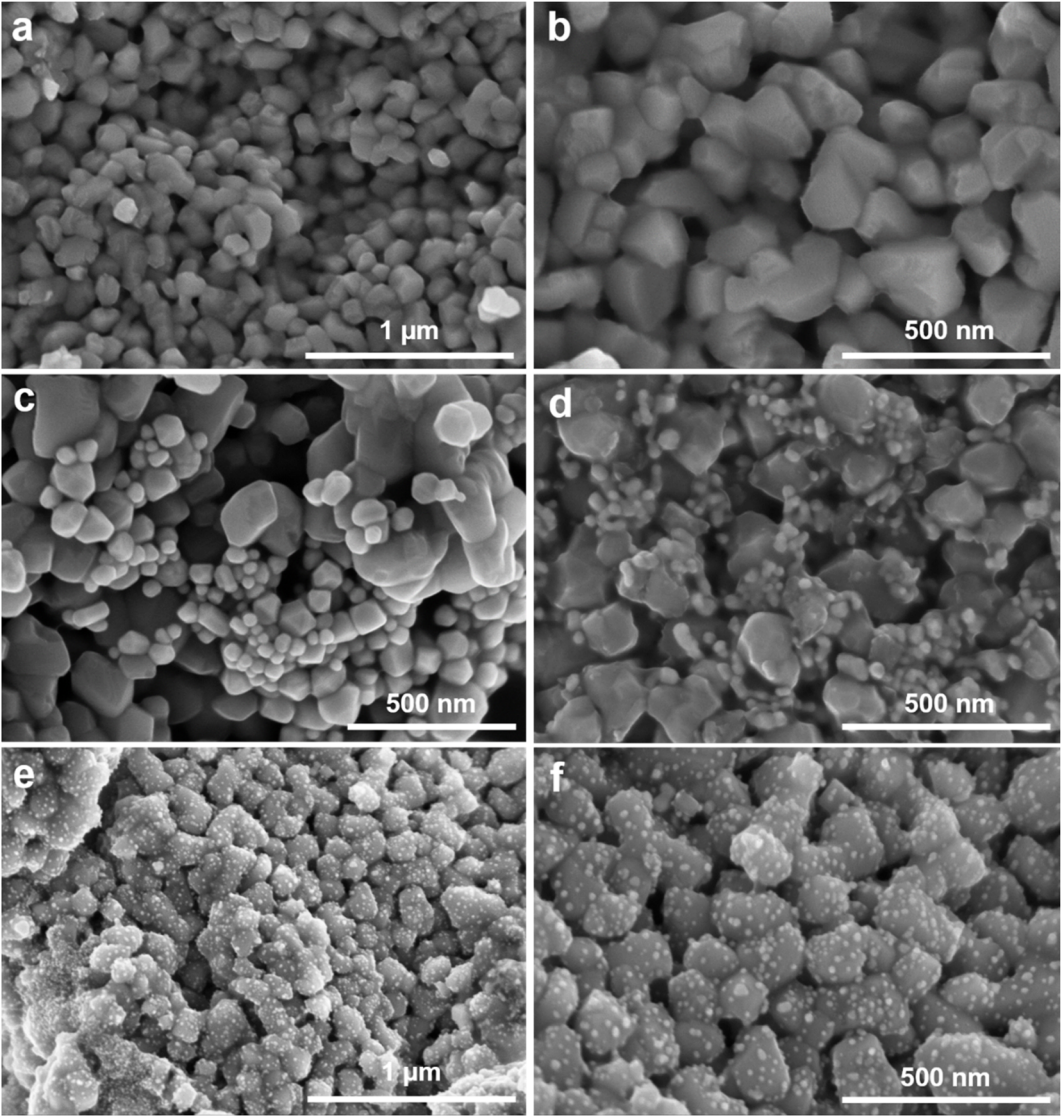
FE-SEM images of TiO_2_ materials (a and b), Ag/TiO_2_ (c and d) and Ag-dotted TiO_2_ nanocomposites (e and f) at different magnifications.

The morphology of the TiO_2_ and Ag-dot TiO_2_ NC materials was investigated using the FE-SEM method, and the results are presented in Fig. 1. Fig. 1a and b show FE-SEM images of the TiO_2_ material synthesized *via* an electrochemical method followed by thermal treatment at 800 °C. It can be observed that the TiO_2_ particles exhibit a generally spherical shape. However, due to annealing at 800 °C, preferential crystal facets of the rutile phase are formed, leading to asymmetric particle morphology. The particle sizes are non-uniform, ranging from approximately 100 to 300 nm. Fig. 1c–f show the FE-SEM images of Ag/TiO_2_ NCs prepared with different CTAB concentrations. Fig. 1c shows the morphology of Ag/TiO_2_ NCs synthesized without CTAB. In this case, TiO_2_ particles similar to those in Fig. 1a and b can be observed, along with the appearance of smaller, brighter-contrast nanoparticles attributed to Ag formed by the reduction of AgNO_3_. However, these Ag nanoparticles are seen to be spatially separated from the TiO_2_ surfaces, exhibiting relatively large sizes and a mild tendency toward aggregation. Fig. 1d displays the Ag/TiO_2_ NCs synthesized with 0.1 M CTAB solution. The TiO_2_ particles maintain their shape and size, while numerous small and relatively uniform Ag nanoparticles with diameters of approximately 40–50 nm are observed near the TiO_2_ surfaces. In addition, a faint coating layer surrounding the TiO_2_ particles can be distinguished and is attributed to the presence of a large number of CTAB molecules. This suggests that an excessive amount of CTAB may hinder the direct interaction between Ag^+^ ions and the TiO_2_ surface, leading to the formation of isolated Ag nanoparticles detached from TiO_2_. Compared to the CTAB-free sample, the Ag nanoparticles obtained in this case exhibit more uniform sizes and reduced aggregation. Fig. 1e and f display the FE-SEM images of Ag/TiO_2_ NCs prepared with 0.01 M CTAB. As observed across all samples, the TiO_2_ structure remains unchanged after the formation of Ag nanoparticles through chemical reduction, with its shape and size well preserved. Notably, when 0.01 M CTAB is used, small Ag nanoparticles with sizes around 20–30 nm are clearly visible, uniformly dispersed across the surface of the TiO_2_ particles. This feature is consistently observed across all examined TiO_2_ particles, where the Ag nanoparticles form discrete dots evenly distributed on the TiO_2_ surface, thus termed Ag-dotted TiO_2_ nanocomposites. These observations indicate that the presence of CTAB at an appropriate concentration (0.01 M) facilitates the electrostatic interaction between Ag^+^ ions and the TiO_2_ surface, promoting localized nucleation and direct growth of Ag on TiO_2_. In contrast, the absence or excessive presence of CTAB disrupts this interaction, resulting in the separate formation of Ag nanoparticles detached from the TiO_2_ surface. Owing to the uniform dispersion of Ag on each TiO_2_ nanoparticle and the intimate interfacial contact between Ag and TiO_2_ achieved at 0.01 M CTAB, these Ag-dot TiO_2_ NCs are expected to exhibit good SERS and PIERS performance compared to the other structures. Therefore, the subsequent characterizations mainly focused on evaluating the properties of the Ag-dot TiO_2_ NCs.

**Fig. 2:**
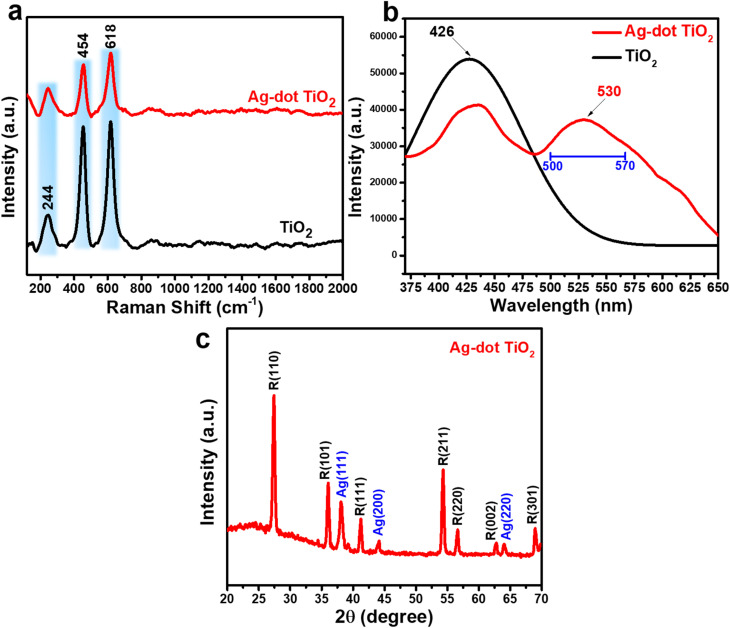
Raman spectra (a) and PL spectra (b) of TiO_2_ and Ag-dotted TiO_2_ nanocomposite materials; XRD pattern of Ag-dotted TiO_2_ nanocomposite materials (c).

Fig. 2a illustrates the Raman spectra of TiO_2_ and Ag-dot TiO_2_ NC materials. For TiO_2_, characteristic scattering peaks are observed at 244, 454, and 618 cm^−1^, corresponding to the E_g_ and A_1g_ vibrational modes of the rutile crystal phase. Similar features are present in the Raman spectrum of the Ag-dot TiO_2_ NCs, with no additional or unexpected peaks detected. This indicates that no foreign or impurity phases were introduced during the chemical reduction process used to incorporate Ag into TiO_2_. Fig. 2b shows the PL spectra of TiO_2_ and Ag-dot TiO_2_ NCs. The TiO_2_ spectrum exhibits a strong emission peak at 426 nm, corresponding to a band gap energy of approximately 2.9 eV – consistent with the rutile phase. Notably, the PL spectrum of Ag-dot TiO_2_ NCs displays significant changes compared to that of pure TiO_2_. While the characteristic 426 nm peak remains, its intensity is reduced. Additionally, a new emission peak emerges at a longer wavelength of approximately 530 nm. This observation suggests the presence of an intermediate energy level within the band gap of the TiO_2_ crystal. Importantly, this intermediate state does not quench the original TiO_2_ emission, but rather reduces its intensity while introducing a new, lower-energy emission band. This implies that the intermediate level does not directly alter the electronic band structure of TiO_2_ (*i.e.*, it does not cause a band gap narrowing), but instead acts as an external electronic state. This state facilitates a new charge-transfer pathway from TiO_2_ to the intermediate level, leading to the observed red-shifted emission band. These results confirm a strong interfacial interaction between TiO_2_ and Ag, which enables efficient charge transfer through a metal/semiconductor transition process. The crystalline properties of the Ag-dotted TiO_2_ NCs were analyzed by XRD analysis, and the corresponding pattern is presented in Fig. 2c. Clearly defined and sharp diffraction peaks are observed at various positions, corresponding to the characteristic diffractions of rutile-phase TiO_2_ and metallic Ag. Specifically, the peaks located at 2*θ* = 27.4°, 36.1°, 41.3°, 54.3°, 56.6°, 62.8°, and 69.0° are indexed to the (110), (101), (111), (211), (220), (002), and (301) crystal planes of rutile TiO_2_, consistent with the standard JCPDS card no. 21-1276. The presence of a highly crystalline rutile phase is reasonable, as the material was thermally annealed at 800 °C, which is also in good agreement with the Raman analysis. In addition, the XRD pattern reveals several diffraction peaks at 2*θ* = 38.1°, 43.3°, and 64.4°, corresponding to the (111), (200), and (220) planes of face-centered cubic (fcc) Ag (JCPDS no. 04-0783). These results confirm that the Ag-dotted TiO_2_ NCs possess high crystallinity for both TiO_2_ and Ag phases.

### 3.2 PIERS sensing performance of Ag-dotted TiO_2_ nanocomposites toward Congo red (a bulky dye)

Congo red (CR) is a large-sized molecule with bulky diazo dye with two sulfonated naphthalene rings linked by azo groups, forming an extended π-conjugated structure. This configuration leads to weak Raman activity due to limited polarizability changes during vibrations. These characteristics make CR a typical weak-Raman-response molecule, challenge for trace detection using conventional SERS substrates. Therefore, CR was selected as a model analyte to investigate the SERS activity of the Ag-dot TiO_2_ NCs in comparison with other Ag/TiO_2_ NCs synthesized at different CTAB concentrations, and to assess the enhancement efficiency of the PIERS effect.

**Fig. 3:**
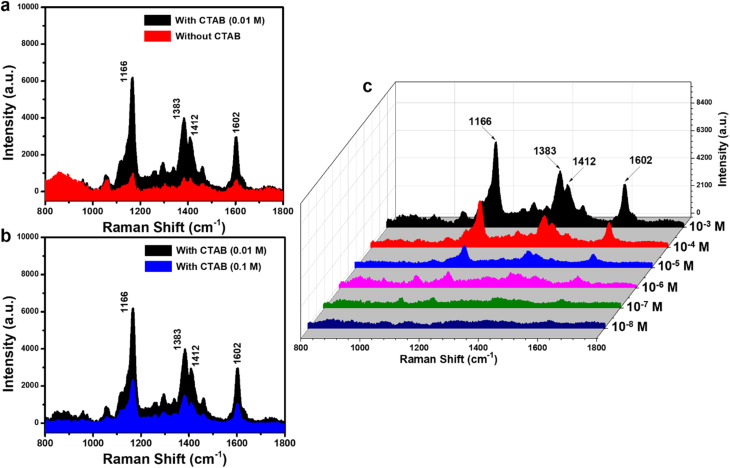
Comparison of the SERS signals obtained from Ag-dotted TiO_2_ NC substrates with those of Ag/TiO_2_ NCs synthesized without CTAB (a) and with an excessive amount of CTAB (b), SERS spectra of CR obtained on Ag-dot TiO_2_ NC substrates without UV pre-irradiation (c).

Initial experiments for CR detection using conventional SERS (without UV pre-irradiation) were conducted on Ag-dot TiO_2_ NC substrates (0.01 M CTAB) in comparison with Ag/TiO_2_ NCs synthesized without CTAB and with an excessive amount of CTAB (0.1 M), and the results are shown in Fig. 3. Fig. 3a shows the SERS spectra of CR (10^−3^ M) obtained from two SERS substrates: Ag-dotted TiO_2_ NCs and Ag/TiO_2_ NCs synthesized without CTAB. The characteristic Raman peaks of CR are clearly detected in both spectra; however, their intensities are markedly stronger and more defined for the Ag-dotted TiO_2_ NCs. This pronounced enhancement can be attributed to the uniform and intimate contact between Ag nanoparticles and the TiO_2_ surface, which facilitates stronger electromagnetic coupling and charge-transfer interactions. In contrast, the Ag/TiO_2_ NCs synthesized without CTAB exhibit irregularly distributed, aggregated Ag particles, leading to weaker SERS signals. Similarly, Fig. 3b demonstrates that the SERS performance of Ag/TiO_2_ NCs synthesized with an excessive CTAB concentration (0.1 M) is significantly lower than that of the Ag-dotted TiO_2_ NCs. These findings confirm that a well-dispersed and uniformly decorated Ag nanoparticle layer on TiO_2_ yields a superior SERS response compared with structures containing nonuniform and isolated Ag particles. Consequently, subsequent SERS measurements for CR detection were conducted using the optimized Ag-dotted TiO_2_ NC substrate. A concentration series of CR ranging from 10^−3^ to 10^−8^ M was directly dropped onto the Ag-dot TiO_2_ NC substrates, and the corresponding SERS spectra were collected (Fig. 3c). At a concentration of 10^−3^ M, characteristic SERS peaks of CR appeared at 1166, 1383, 1412, and 1602 cm^−1^. The peak at 1166 cm^−1^ corresponds to the C–N= stretching vibration, while the peaks at 1383 cm^−1^ and 1412 cm^−1^ are attributed to N=N stretching.^24,25^ The 1602 cm^−1^ peak is assigned to the aromatic C–C stretching vibration in the molecular structure of CR.^24,25^ These characteristic peaks gradually diminished as the CR concentration decreased. At 10^−7^ M, the CR specific peaks remained detectable but exhibited very weak intensity, and they completely disappeared at 10^−8^ M. Although SERS is a highly sensitive technique, the detection limit for CR was only down to 10^−7^ M, which is below expectations. Furthermore, the weak SERS signals even at higher concentrations indicate that CR is a molecule with intrinsically weak Raman response.

**Fig. 4:**
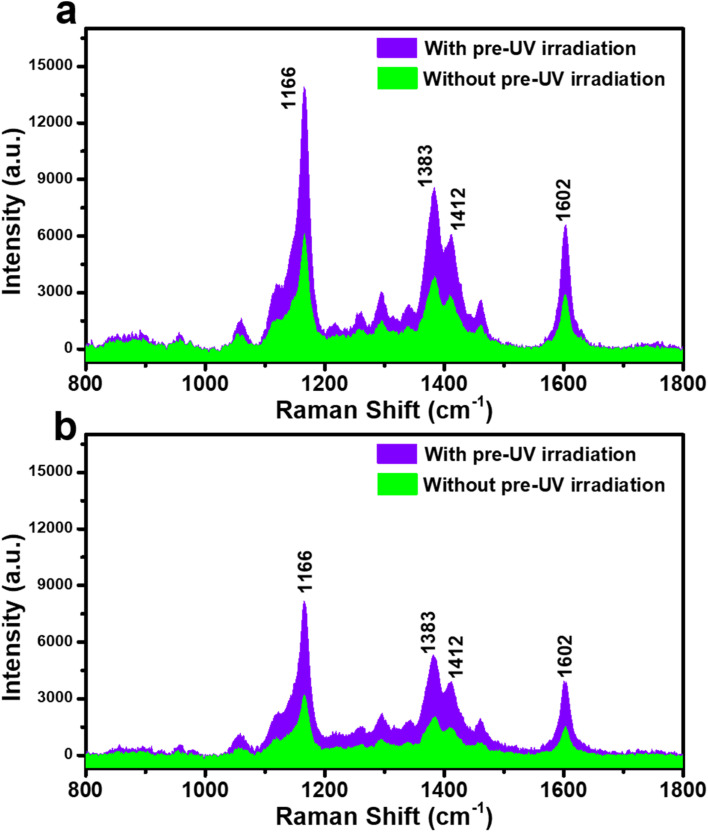
Comparison between conventional SERS (without UV pre-irradiation) and PIERS (with UV pre-irradiation) spectra obtained on the Ag-dot TiO_2_ NCs substrate for CR concentrations of 10^−3^ M (a) and 10^−4^ M (b).

**Fig. 5:**
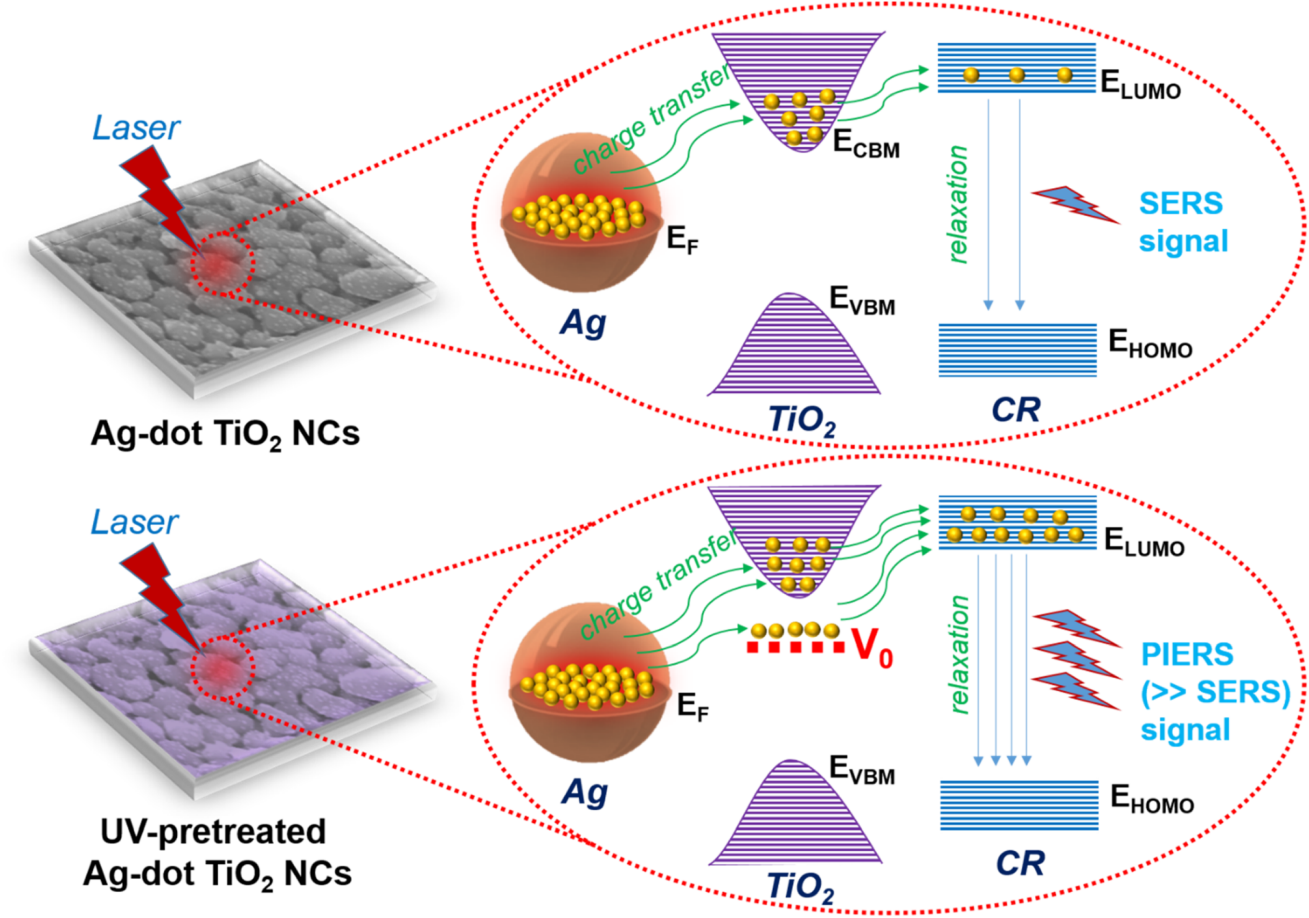
Schematic illustration of the SERS and PIERS mechanisms involved in CR detection using the Ag-dot TiO_2_ NC substrates.

The PIERS effect of the Ag-dot TiO_2_ NCs substrate is expected to enhance the sensing performance toward CR compared to conventional SERS. By introducing a pre-irradiation step using UV light at 365 nm into the SERS measurement protocol, the PIERS behavior of the Ag-dot TiO_2_ NC substrate toward CR was evaluated at concentrations of 10^−3^ M and 10^−4^ M, as illustrated in Fig. 4 and compared with conventional SERS. It can be observed that the PIERS spectra of CR at both concentrations exhibit characteristic Raman peaks identical in position to those in the SERS spectra, indicating no peak shift. Remarkably, the signal intensity of CR significantly increases under PIERS conditions, with sharper and more intense peaks observed at both 10^−3^ M (Fig. 4a) and 10^−4^ M (Fig. 4b) compared to conventional SERS. This enhancement demonstrates that the UV pre-irradiation of the Ag-dot TiO_2_ NCs substrate contributes effectively to signal amplification. This enhancement is attributed to a charge-transfer-based mechanism involving the three-component system of Ag, TiO_2_, and CR under PIERS conditions. Fig. 5 illustrates the proposed enhancement mechanism of PIERS in comparison with conventional SERS. In the absence of UV pre-irradiation, SERS signals are generated through the synergistic effect of the EM mechanism from the Ag nanoparticles and the CM enhancement arising from the Ag/TiO_2_ junction. The incorporation of TiO_2_ in the SERS substrate facilitates charge transfer from Ag to CR. Upon laser excitation, LSPR in Ag nanoparticles generates energetic hot charges, which can transfer to the conduction band minimum (E_CBM_) of TiO_2_ and subsequently to the CR molecules.^26–28^ Under PIERS conditions, however, the UV pre-irradiation with high-energy photons temporarily removes surface oxygen species from TiO_2_, thereby creating transient oxygen vacancies (V_0_) within the bandgap.^29^ These V_0_ states form a temporary mid-gap energy level approximately 0.7 eV below the E_CBM_.^30^ The presence of these intermediate levels significantly promotes more efficient charge transfer from Ag to TiO_2_ and ultimately to CR molecules, resulting in substantially enhanced Raman signals.

**Fig. 6:**
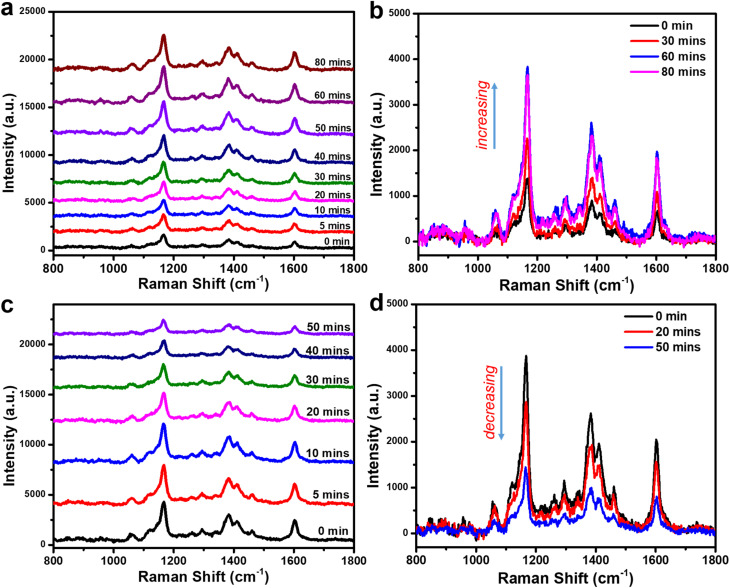
Sensing signals of CR collected on Ag-dot TiO_2_ NC substrates at different UV irradiation durations (a and b) and at various recovery time points after irradiation (c and d).

The signal enhancement capability of PIERS is strongly influenced by the presence of oxygen vacancies on the TiO_2_ surface, which are generated under UV irradiation. Consequently, the intensity of the PIERS signal is expected to correlate closely with the density of oxygen vacancies on the TiO_2_ surface. This density can be modulated by varying the UV exposure time of the Ag-dot TiO_2_ NC substrate. To investigate this effect, the experiments were conducted to examine how the PIERS signal intensity evolves with different UV irradiation durations. The results are presented in Fig. 6a and b. Specifically, the substrates were exposed to UV light for 0 (no irradiation), 5, 10, 20, …, and 80 minutes. The obtained data reveal a significant variation in signal intensity depending on the irradiation duration. At short exposure times (5 and 10 minutes), the SERS signal of CR remains nearly unchanged compared to the unirradiated sample. However, for longer durations (20, 30, 40, 50, and 60 minutes), a progressive increase in the intensity of CR's characteristic Raman peaks is observed. This trend is more clearly illustrated in Fig. 6b, where the intensities at 0, 30, and 60 minutes are compared. These results suggest that prolonged UV exposure increases the number of oxygen vacancies on the TiO_2_ surface, thereby enhancing the PIERS signal of CR. Interestingly, further extending the irradiation time to 80 minutes does not lead to a noticeable improvement in signal intensity compared to the 60-minute mark. This plateau indicates a saturation point in the generation of oxygen vacancies, beyond which additional UV exposure does not contribute further to signal enhancement. Therefore, a UV irradiation time of 60 minutes was selected as the optimal condition for subsequent PIERS experiments, balancing both enhancement efficiency and practical experimental duration.

The recovery dynamics of oxygen vacancies on the TiO_2_ surface represent an important factor influencing the PIERS effect.^31^ Upon cessation of UV pre-irradiation, ambient oxygen atoms can gradually refill the vacancies, diminishing the enhancement effect. To investigate this, PIERS signals of CR were recorded at various time intervals following the end of pre-irradiation, as shown in Fig. 6c and d. A clear decrease in signal intensity was observed with increasing recovery time, indicating progressive oxygen vacancy recombination. After approximately 50 minutes, the PIERS signal returned to the baseline level of the non-irradiated substrate, suggesting that the photo-induced oxygen vacancies had fully recovered. Notably, the strongest PIERS signal was detected immediately after UV exposure ended, and this time point was chosen for all subsequent sensing measurements. These results highlight the critical role of oxygen vacancies in PIERS enhancement, as both the increase in signal with longer UV exposure and the decrease during recovery correlate directly with oxygen vacancy dynamics on the Ag-dot TiO_2_ NC substrate.

**Fig. 7:**
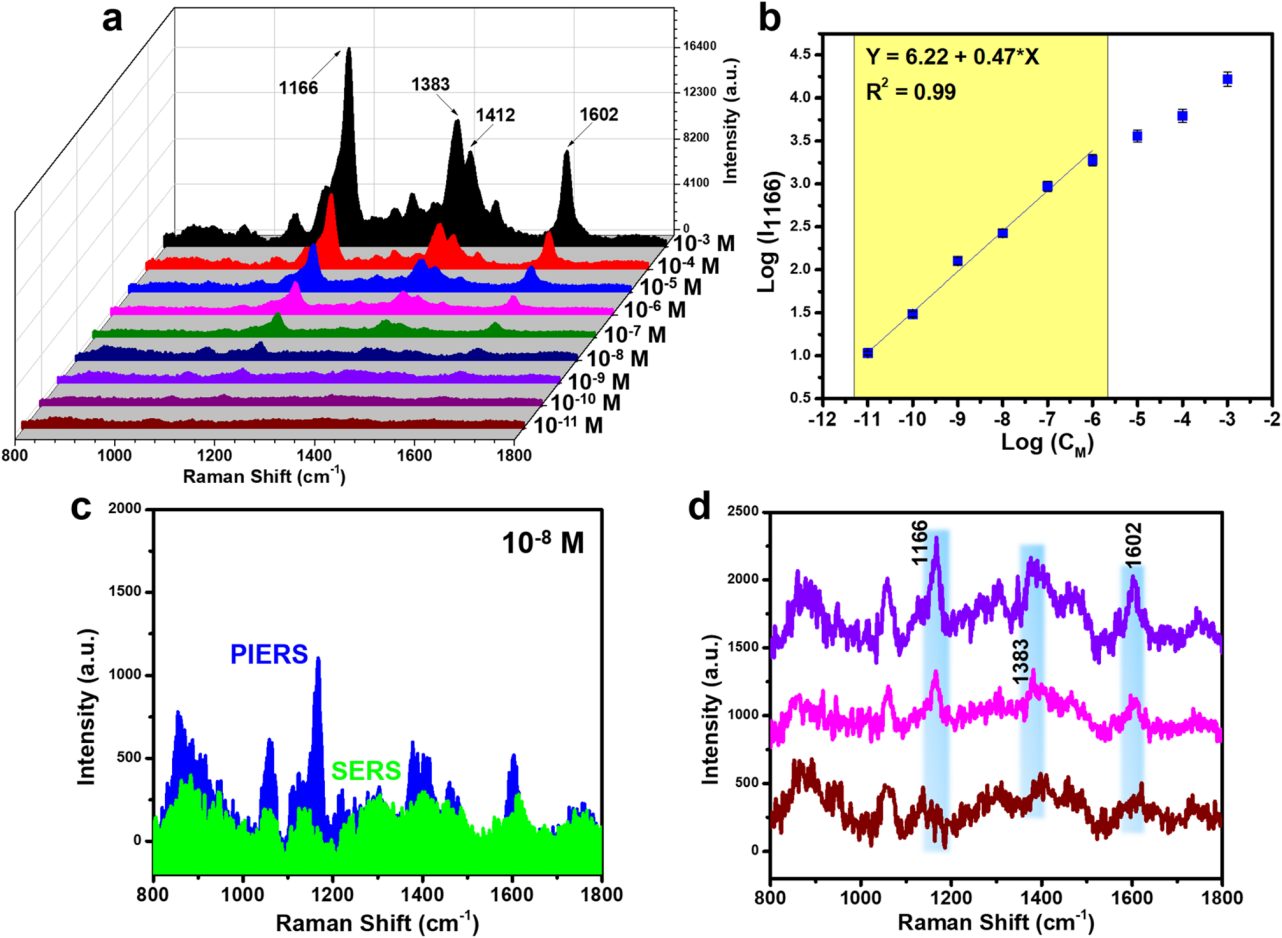
PIERS spectra of CR on Ag-dot TiO_2_ NC substrate in the concentration range of 10^−3^–10^−11^ M (a); linear relationship between CR concentration and signal intensity *via* logarithmic function (b); comparison of sensing signals for CR at 10^−8^ M in PIERS and SERS (c); PIERS spectra of CR on Ag-dot TiO_2_ NCs at concentrations of 10^−9^, 10^−10^, and 10^−11^ M (d).

Fig. 7 illustrates the PIERS sensing performance of Ag-dot TiO_2_ NCs toward CR molecules under the optimized experimental conditions described above. A wide concentration range from 10^−3^ M to 10^−11^ M was applied to the UV pre-irradiated Ag-dot TiO_2_ NC substrates. The corresponding PIERS signals at these concentrations are presented in. Clearly, characteristic Raman peaks of CR are well-resolved and intense across the range of 10^−3^ to 10^−7^ M. Notably, at 10^−8^ M, the PIERS signal of CR remains clearly distinguishable, whereas in the conventional SERS spectrum, the characteristic peaks are almost entirely obscured by background noise (Fig. 7c). Based on the difference in signal intensities, the enhancement factor between PIERS and standard SERS was calculated, showing a maximum enhancement of 8.9-fold at 10^−7^ M. This enhancement outperforms previously reported PIERS substrates, which typically show improvements of only 3 to 7-fold over conventional SERS.^19,20,32,33^ A linear relationship between CR concentration and PIERS signal intensity was established using the peak at 1166 cm^−1^, yielding the regression equation *y* = 6.22 + 0.47 × *x*, where *x* and *y* represent the logarithms of CR concentration and signal intensity, respectively (Fig. 7b). Using this calibration curve and the LOD calculation method outlined in Section 2.5, the detection limit (LOD) of the PIERS technique on Ag-dot TiO_2_ NCs was determined to be as low as 5.32 × 10^−11^ M. Table 1 Compares the CR detection performance of various SERS substrates and techniques. While conventional SERS techniques, even when implemented on sophisticated substrates, generally achieve detection limits around 10^−8^ M, the use of PIERS on Ag-dot TiO_2_ NCs in this study significantly enhances sensitivity, achieving an ultralow LOD of 10^−11^ M.

**Table 1:** Comparison of CR detection performance across various substrates and between conventional SERS and PIERS techniques (CC: carbon cloth; L-MOF: leaf-like metal–organic framework; F–Ag: flower-like Ag nanoparticles)

Substrate	Technique	LOD (M)	Ref.
Cu dendritic nanostructures	SERS	1 × 10^−7^	34
Ag nanoparticles	SERS	∼1 × 10^−8^	35
Ag nanostars	SERS	4.02 × 10^−8^ (28 ppb)	36
CC-L-MOF@F–Ag	SERS	1.3 × 10^−8^	37
Ag-dot TiO_2_ NCs (without pre-UV irradiation)	SERS	1 × 10^−8^	This work
Ag-dot TiO_2_ NCs (with pre-UV irradiation)	PIERS	5.32 × 10^−11^	This work

**Fig. 8:**
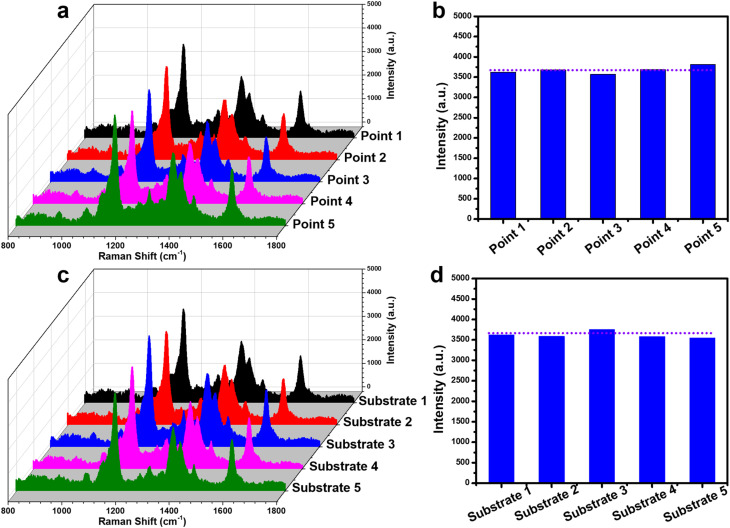
Evaluation of repeatability of the PIERS experiment for detecting CR at a concentration of 10^−5^ M by collecting signals at five different positions on the same Ag-dot TiO_2_ NCs substrate (a and b); and evaluation of reproducibility of the PIERS experiment for detecting CR at a concentration of 10^−5^ M by collecting signals from five independently prepared Ag-dot TiO_2_ NCs substrates (c and d).

The reliability of the PIERS experiment on the Ag-dot TiO_2_ NC substrate was also evaluated through the parameters of repeatability and reproducibility. To assess the repeatability of PIERS, the sensing signals of CR at a concentration of 10^−5^ M were collected at five different locations on the same Ag-dot TiO_2_ NC substrate, and the results are shown in Fig. 8a. It can be seen that the characteristic peaks and their intensities exhibit high similarity across all five random positions. Quantitatively, the 1166 cm^−1^ peak was selected to calculate the relative standard deviation (RSD), resulting in a value of 6.27% (Fig. 8b). The reproducibility parameter was evaluated by measuring the sensing signals of CR at a concentration of 10^−5^ M on five Ag-dot TiO_2_ NC substrates prepared in five independent batches. The results presented in Fig. 8c and d indicate high reproducibility of the PIERS experiment on Ag-dot TiO_2_ NCs, with an RSD value of 7.22%. It can be concluded that the RSD values for both repeatability and reproducibility are below 10%, indicating high reliability of the PIERS experiment on Ag-dot TiO_2_ NCs.

### 3.3 Application of Ag-dotted TiO_2_ nanocomposite PIERS substrate for other analytes: thiram (a pesticide) and urea (a small biomarker)

**Fig. 9:**
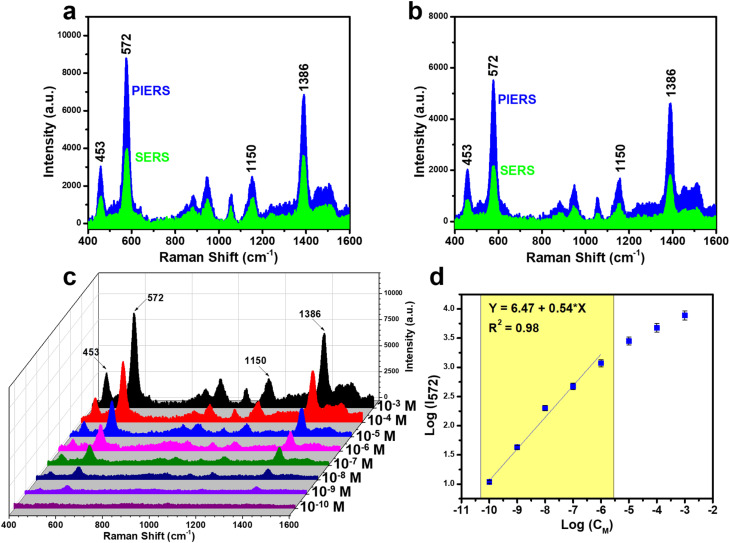
Comparison of thiram sensing signals between PIERS and conventional SERS at concentrations of 10^−3^ M (a) and 10^−4^ M (b); PIERS spectra of thiram in the concentration range of 10^−3^–10^−10^ M (c); and the linear relationship between thiram concentration and signal intensity on a logarithmic scale (d).

The high sensing performance of PIERS on the Ag-dot TiO_2_ NCs substrate was further evaluated using two additional molecular targets: thiram, representing the pesticide group, and urea, representing small biomarker molecules. Both of these analytes are of significant practical importance: thiram is a commonly used fungicide that must be strictly controlled at trace levels in agricultural products, while urea is an essential biomarker in biomedical diagnostics. The PIERS sensing performance for thiram is illustrated in Fig. 9. As shown in Fig. 9a and b, the Raman signal intensity obtained *via* the PIERS method was compared with that of conventional SERS for thiram at concentrations of 10^−3^ M and 10^−4^ M. It can be observed that the characteristic peaks of thiram at 453, 572, 1150, and 1386 cm^−1^ appear with significantly enhanced intensity and sharper features in the PIERS case. A wide concentration range of thiram, from 10^−3^ M to 10^−10^ M, was prepared and analyzed using PIERS on the Ag-dot TiO_2_ NC substrate to determine the LOD value. As shown in Fig. 9c, characteristic peaks of thiram remained clearly detectable even at low concentrations of 10^−8^ and 10^−9^ M, and only disappeared at 10^−10^ M. A linear relationship between the concentration and Raman intensity of thiram was established based on the 572 cm^−1^ peak, resulting in a linear regression equation of *y* = 6.47 + 0.54 × *x* (Fig. 9d). The calculated LOD was 1.89 × 10^−10^ M, which is an extremely low level and well meets the safety threshold for the maximum allowable thiram content in food products.

**Fig. 10:**
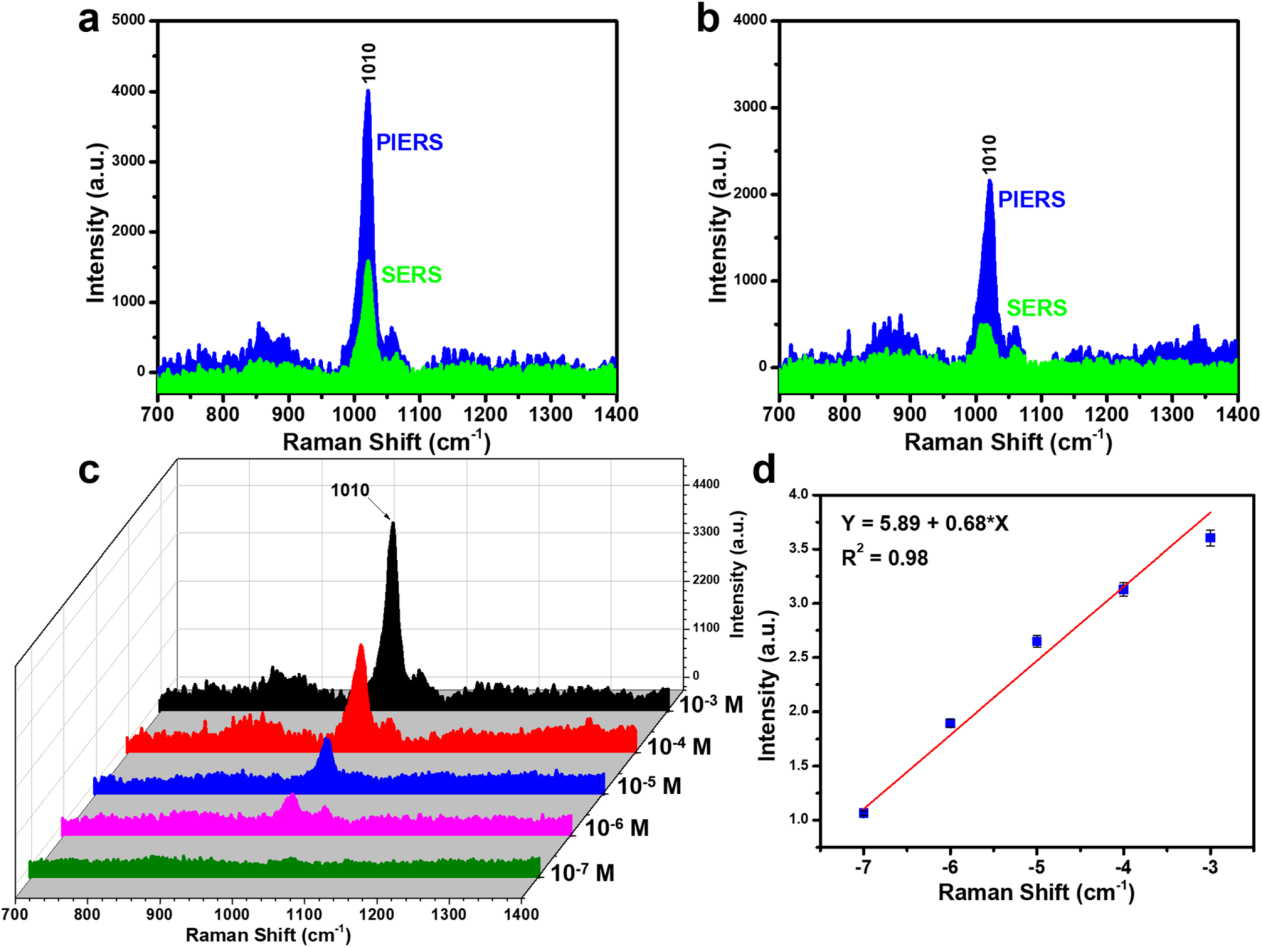
Comparison of urea sensing signals between PIERS and conventional SERS at concentrations of 10^−3^ M (a) and 10^−4^ M (b); PIERS spectra of urea in the concentration range of 10^−3^–10^−10^ M (c); and the linear relationship between urea concentration and signal intensity on a logarithmic scale (d).

For urea molecules, conventional SERS sensors face significant challenges in detection, with the typical LOD only reaching 10^−3^ to 10^−4^ M.^38,39^ The application of PIERS on Ag-dot TiO_2_ NC substrates markedly enhances the sensing performance for this important biomarker, which is associated with various severe diseases. Fig. 10a and b compare the urea detection efficiency between PIERS and conventional SERS. The characteristic peak at 1010 cm^−1^, corresponding to the C–N stretching mode in the urea molecular structure, appears prominently with high intensity under PIERS conditions.^40^ In contrast, this peak shows very low intensity even at a relatively high concentration of 10^−4^ M in the case of conventional SERS, highlighting the superior signal enhancement capability of PIERS for urea detection. Fig. 10c demonstrates the PIERS sensing performance for urea across a concentration range from 10^−3^ to 10^−7^ M. The urea signal remains distinguishable even at 10^−6^ M. A calibration curve with the linear equation *y* = 5.89 + 0.68 × *x* yields a LOD as low as 2.32 × 10^−7^ M, representing a significant improvement over conventional SERS (Fig. 10d). With such enhanced sensing capability, the PIERS technique based on Ag-dot TiO_2_ NCs shows strong potential for the trace detection of urea. Despite the distinct molecular structures and interaction behaviors of CR, thiram, and urea with the Ag-dot TiO_2_ NCs, all three analytes exhibited pronounced PIERS enhancement under identical experimental conditions. This observation confirms that the Ag-dot TiO_2_ NC substrate facilitates photo-induced charge transfer between TiO_2_ and the adsorbed molecules in a broad and non-specific manner. The results indicate that the enhancement mechanism is primarily governed by the dynamic modulation of oxygen vacancies and interfacial charge transfer, rather than by specific molecular configurations. Therefore, the developed PIERS platform demonstrates universal applicability toward a wide range of molecular species, including those with intrinsically weak Raman responses, as the photo-induced surface activation effectively enhances molecular polarizability and Raman cross-sections in a generalizable fashion.

## 4 Conclusions

We have demonstrated the superior sensing performance of photo-induced enhanced Raman spectroscopy (PIERS) on Ag-dotted TiO_2_ nanocomposite (Ag-dot TiO_2_ NCs) substrates, significantly surpassing that of conventional SERS. This enhancement is achieved through a simple UV pre-irradiation process, effectively boosting signal response across a diverse range of analytes, particularly those exhibiting inherently weak Raman activity. The uniform decoration of Ag nanoparticles on rutile-phase TiO_2_ nanoparticles creates consistent and intimate metal-semiconductor contact sites throughout the substrate, which is critical for the enhanced PIERS effect. Under optimal conditions, the PIERS platform delivered an impressive 8.9-fold signal enhancement compared to conventional SERS. Notably, it enabled ultralow detection limits for several representative analytes: 5.32 × 10^−11^ M for Congo red – a bulky organic dye with weak Raman scattering; 2.32 × 10^−7^ M for urea – a small biomolecule typically difficult to detect using standard SERS; and 1.89 × 10^−10^ M for thiram – a pesticide of regulatory concern in food safety. In addition to high sensitivity, the system exhibited excellent analytical reliability, with repeatability and reproducibility yielding relative standard deviations (RSDs) below 10%. These results confirm that PIERS on Ag-dot TiO_2_ NCs provides an effective solution to key limitations of conventional SERS when applied to weak-Raman-response molecules. Overall, this work highlights the great potential of PIERS using Ag-dot TiO_2_ NCs for ultrasensitive and reliable trace detection in critical application areas such as environmental monitoring, food safety, and biomedical diagnostics.

## Author contributions

Q. D. Mai: conceptualization, methodology, investigation, formal analysis, data curation, supervision, writing – original draft; D. T. H. Trang: formal analysis, investigation, validation; V. T. L. Na: validation, investigation; T. N. Bach: validation, investigation; O. V. Hoang: validation, investigation; N. T. T. Tuyen: validation, investigation; N. D. Hung: validation, investigation; A. T. Pham: methodology, supervision; A. T. Le: conceptualization, methodology, supervision, project administration, writing – review & editing.

## Conflicts of interest

The authors declare that they have no known competing financial interests or personal relationships that could have appeared to influence the work reported in this paper.

## Data Availability

The data supporting the findings of this study are available from the corresponding author upon reasonable request. All experimental data, including the characterization of the Ag-dotted TiO_2_ nanocomposite substrates and the detection results for Congo red, thiram and urea molecules, are included in the manuscript.
